# Thymoma with an isolated splenic metastasis eight years after extended thymectomy: a case report

**DOI:** 10.1186/s12885-018-5165-0

**Published:** 2018-12-13

**Authors:** Yuichi Aoki, Atsushi Miki, Tomoyuki Nakano, Hideki Sasanuma, Yasunaru Sakuma, Hisanaga Horie, Yoshinori Hosoya, Noriyoshi Fukushima, Alan Kawarai Lefor, Naohiro Sata

**Affiliations:** 1 Department of Surgery, Jichi Medical University, 3311-1 Yakushiji, Shimotsuke, Tochigi Japan; 2 Department of Diagnostic Pathology, Jichi Medical University, 3311-1 Yakushiji, Shimotsuke, Tochigi Japan

**Keywords:** Spleen, Thymic tumor, Metastasis, Laparoscopic splenectomy

## Abstract

**Background:**

Thymomas are typically slow-growing tumors and AB type thymomas are considered no/low risk tumors with a better prognosis. Extra-thoracic metastases are extremely rare. To the best of our knowledge, no patient with an isolated splenic metastasis from a thymoma has been reported. We report a patient who underwent laparoscopic splenectomy for a slow-growing, isolated splenic metastasis, eight years after thymectomy.

**Case presentation:**

The patient is a 78-year-old man. Eight years previously, the patient underwent extended thymectomy and postoperative radiation therapy for a thymoma. Five years after thymectomy, a nodule appeared in the spleen, and the lesion enlarged gradually for three years thereafter. The patient was referred for further examination and treatment. Computed tomography scan showed a sharply circumscribed 50 mm tumor slightly hypodense and heterogeneous lesion in the spleen. On T2-weighted images on Magnetic Resonance Imaging, the tumor had high intensity, equivalent to or slightly lower than that on T1-weighted images, and no decrease on diffusion-weighted images. The tumor was multinodular and showed a low-signal spoke-wheel sign in the margin, enhanced gradually in the dynamic study. Positron emission tomography-CT scan, showed relatively low accumulation. Surgical resection was undertaken, and pathological examination showed metastatic thymoma. The patient is without recurrence and has no other symptoms three years after splenectomy.

**Conclusions:**

This is the first report of an isolated splenic metastasis from a thymoma. Further cases are needed to standardize this surgery for such lesions.

## Background

Thymomas are typically slow-growing tumors and type AB thymomas are considered no/low risk tumors with a generally better prognosis. Although recurrence and metastasis of type AB thymoma have been reported, their progression commonly occurs in patients with direct invasion or pleural dissemination, and extra-thoracic metastases are extremely rare. To the best of our knowledge, no patient with an isolated splenic metastasis has been reported in the literature. Here we report a patient who underwent laparoscopic splenectomy for a slow-growing, isolated splenic metastasis, eight years after undergoing extended thymectomy for a thymoma.

### Case presentation

The patient is a 78-year-old man referred to our department for splenomegaly seen on abdominal computed tomography (CT) scan. Eight years previously, the patient underwent extended thymectomy and postoperative radiation therapy for a thymoma (WHO classification, AB type; Masaoka classification/TNM classification, stage II) with subsequent follow-up. Five years after resection, a low density, isolated nodule in the spleen was seen on CT scan. Although follow-up continued for three years thereafter, the lesion was noted to continually increase in size, and the patient was then referred for further examination and treatment.

The patient underwent appendectomy for acute appendicitis at the age of 23 years, treatment for hypertension since he was 61 years old, extended thymectomy at age 71 years, and laparoscopic left hemicolectomy for cancer of the descending colon (stage I) at age of 73 years of age. The remainder of the history and physical examination were unremarkable, except for healed surgical scars from the previous thymectomy and the laparoscopic left hemicolectomy. Laboratory studies showed no abnormalities. Serum tumor marker levels were within normal limits (CEA 1.9 ng/ml, CA19–9 12 U/ml, SCC 1.5 ng/ml, CYFRA 2.0 ng/ml, PSA 1.29 ng/ml).

**Fig. 1:**
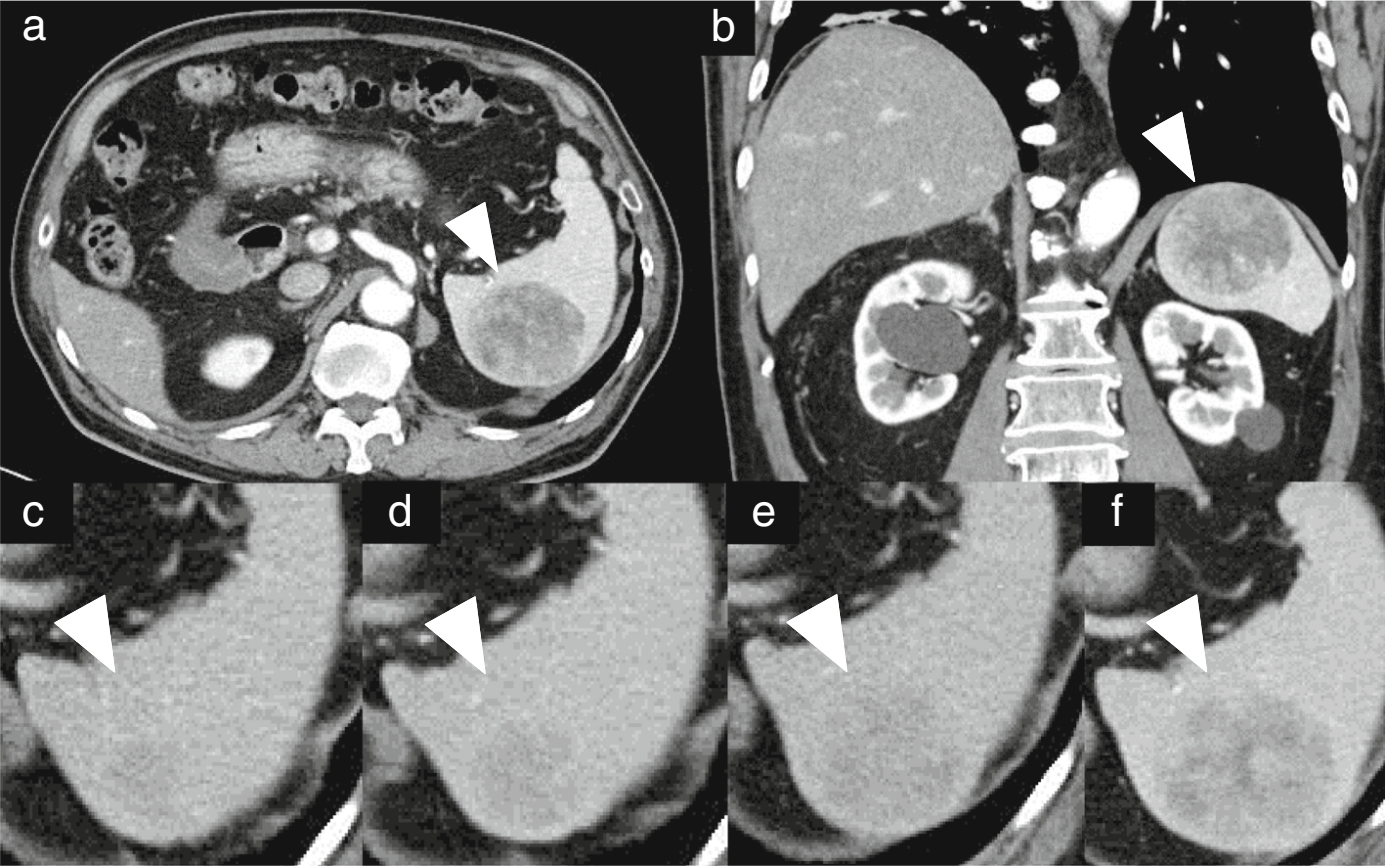
**a** and **b**: Computed tomography imaging shows a sharply circumscribed 50 mm tumor with slightly decreased uptake and a heterogeneous appearance in the spleen. **c**-**f**: A nodule appeared five years after thymectomy and enlarged gradually for three years thereafter. **d**: Six years after thymectomy. **e**: Seven years after thymectomy. **f**: Eight years after thymectomy. Arrows in all panels point to the metastatic lesion in the spleen

**Fig. 2:**
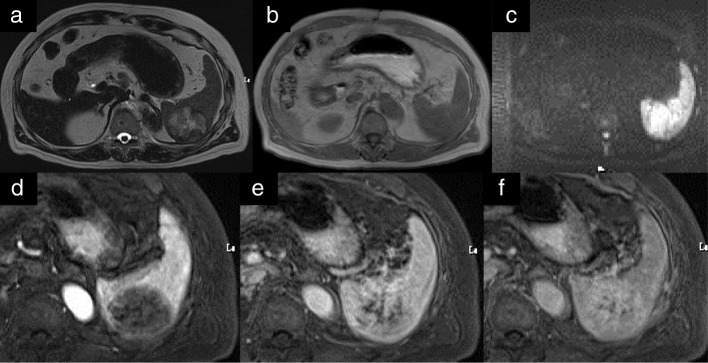
**a-c**: The tumor has high intensity on magnetic resonance imaging (MR) T2WI, equivalent to or slightly lower than that in MRI-T1WI, and no decrease in diffusion. **d-f**: The tumor is multinodular with a low-signal spoke-wheel sign in the margin, enhanced gradually in the dynamic study

**Fig. 3:**
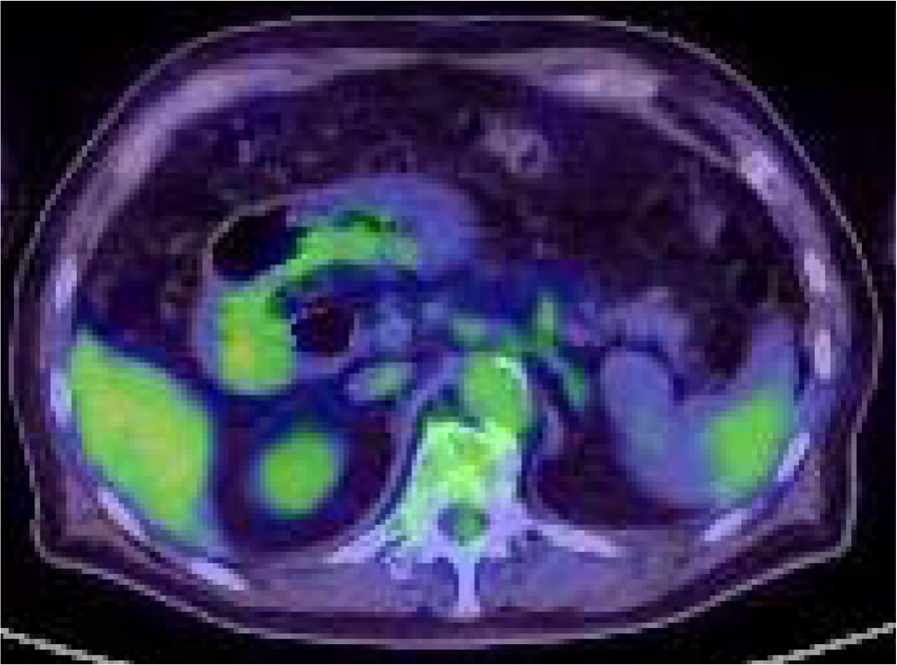
Whole-body positron-emission tomography/computed tomography (PET/CT) imaging. The tumor shows relatively low uptake

CT scan showed a sharply circumscribed 50 mm tumor slightly hypodense and heterogeneous enhancing lesion in the spleen with no intraperitoneal lymphadenopathy (Fig. 1). On Magnetic Resonance Imaging (MRI), T2-weighted images, the tumor had high intensity, equivalent to or slightly lower than that on T1-weighted images, and no decrease on diffusion-weighted images. The tumor was multinodular and had a low-signal spoke-wheel sign in the margin, enhanced gradually on the dynamic study (Fig. 2). Follow-up CT scan showed the tumor gradually increased in size over three years. On positron emission tomography (PET)-CT, the tumor had relatively low accumulation (Fig. 3).

Based on these diagnostic imaging studies, we considered metastatic thymoma, hemangioma or lymphangioma with bleeding and organization, and sclerosing angiomatoid nodular transformation in the differential diagnosis. Surgical resection was then recommended to the patient and undertaken. The lesion was visually confirmed intraoperatively. No extracapsular invasion was observed. A laparoscopic splenectomy without lymphadenectomy was performed.

**Fig. 4:**
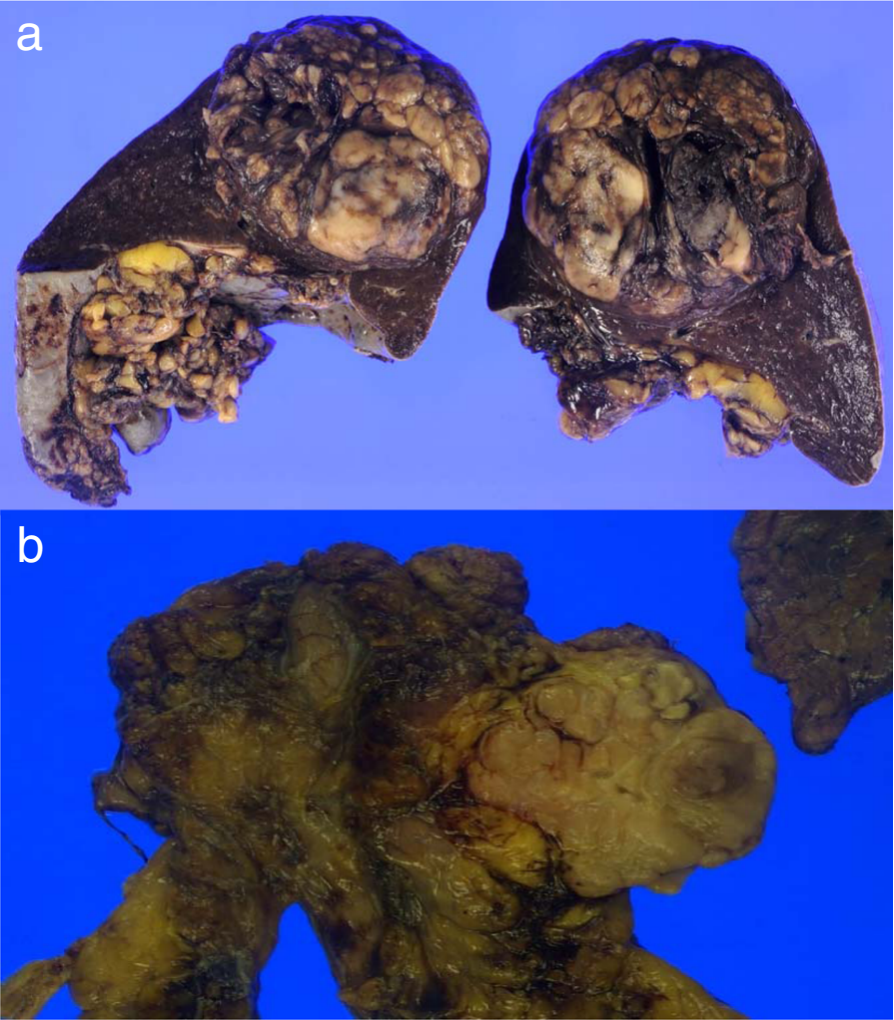
Gross pathology images. **a**: Splenic tumor, **b**: Previously resected thymoma

**Fig. 5:**
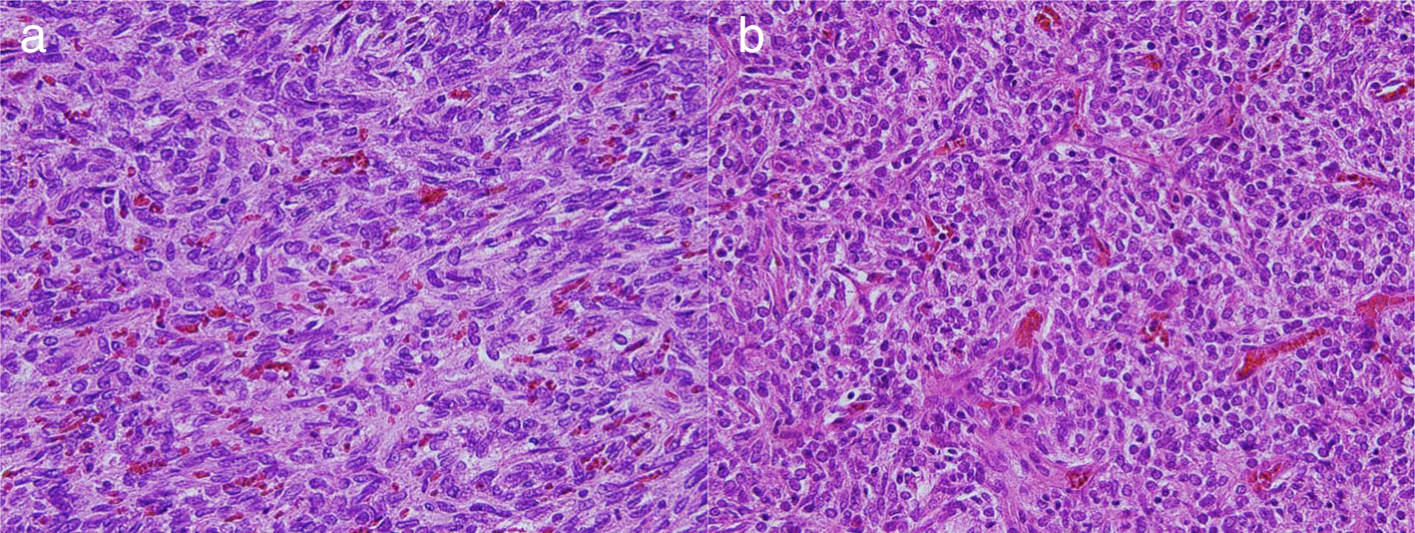
Microscopic images (hematoxylin and eosin stain, X100). The morphology of the cells in the splenic mass is similar to the resected thymoma. **a**: The tumor in the spleen, **b**: The resected thymoma

Pathological examination showed that the spleen was 12 × 7 × 6 cm in size. A lobulated medullary tumor was seen, 6 × 5 × 4.5 cm in size, yellow-brown in color on cut surface (Fig. 4). The capsular structure of the spleen was maintained. Histologically, spindle to slightly epithelioid-shaped cells homogeneous in size with abundant nuclear divisions were growing in a medullary manner with blood vessels. The lesion was morphologically similar to the previously resected thymoma (Fig. 4). Pancytokeratin and p63 were positive, while CD5 and c-Kit were negative histochemically. Vascular endothelium markers (CD31/CD34/factor VIII) were negative. TdT-positive lymphocytes were not discovered on immunostaining. Based on its histological appearance, the splenic mass was unlikely to be a primary splenic tumor or related to a hematological disease. The lesion was not consistent with a metastasis from the previous colon cancer. The histological appearance of the tumor was similar to the previously resected thymoma, and the final pathological diagnosis was metastatic thymoma (Fig. 5).

The patient is now three years status post resection of the spleen and is followed every three months. He is in good condition without recurrence or any other symptoms.

## Discussion

Thymomas are anterior mediastinal tumors that usually arise in 40–70 year old patients. Thymomas are also associated with myasthenia gravis, pure red cell aplasia, and hypo-γ-globulinemia. Although thymomas constitute about one half of malignant tumors in the anterior mediastinum, they are rare, with a frequency of 1.5 per 1 million people [1–5]. The 5-year survival rate is approximately 78%.

Typical progression of thymoma is direct invasion and dissemination into the thoracic cavity, then extra-thoracic distant metastases that arise through hematogenous spread are considered uncommon [6]. Lewis et al. studied 283 patients with thymoma and reported that extra-thoracic distant metastases in only eight patients (3%), all of whom had widespread metastatic disease [7]. Huang et al. discussed 15 patients with recurrence among 97 patients who had undergone resection of thymoma and reported that only two patients had distant metastases to the lung, and no patients had extra-thoracic metastases [8]. Although some patients with extra-thoracic metastases from a thymoma such as a liver, pancreas, ovarian, breast, brain, spine have been reported, but an isolated splenic metastasis has not been reported to date [9–14]. There are a few previous reports of splenic metastases from thymic carcinomas [15, 16].

Splenic tumors are divided into two major classes, including metastatic and primary tumors. Most metastatic splenic tumors originate from hematological diseases, of which leukemia and malignant lymphoma account for approximately two-thirds. Splenic metastases from non-hematologic malignancies occur as part of widespread systemic disease in the end-stage of the disease usually, and an isolated splenic metastasis is quite rare [17]. Autopsy studies in patients who died of malignancies report metastases of malignant tumors to the spleen in 0.3–7.1% of cases, with malignant melanoma, ovarian cancer, breast cancer, and lung cancer reported most often [18, 19]. Some reports regarding metastases from colorectal cancer, gastric cancer, uterine cancer, ovarian cancer and pancreatic neuroendocrine tumor have been recently documented, with results suggesting that advances in chemotherapy and other systemic treatments have affected the long-term survival of patients with malignant tumors [20–26]. The reason that splenic metastases are rare in patients with non-hematologic malignancies is unclear, but several hypotheses have been suggested. The spleen has few afferent lymphatic vessels and the acute angle of the splenic artery leaving the celiac artery may prevent large clumps of tumor cells from gaining access to the spleen. Rhythmic contractions of the spleen forcing blood flow from the sinusoids to the splenic veins may prevent tumor fixation. In addition, the spleen includes large numbers of lymphocytes and macrophagocytes which may allow immunologic inhibition of the induction and growth of tumor cells [15]. Marymont et al. demonstrated that splenic metastases result from the splenic artery route, splenic vein route, and lymphatic route, and are seen in the venous sinusoids and /or red pulp, supporting a hematogenous origin [17]. Sakuma et al. suggested that congestion in the splenic vein may result in development of a splenic metastasis [24]. Although we suspect a hematogenous origin for the metastasis in this patient, there were no vascular abnormalities and no invasion in the splenic artery or vein.

There are benign primary tumors of the spleen which include hemangiomas and hamartomas. In addition, primary malignant tumors of the spleen can include sarcomatous lesions, because the spleen is of mesodermal origin. Splenic primary neoplastic lesions include malignant lymphoma, hemangioendothelioma, lymphatic sarcoma, fibrosarcoma, and malignant fibrous histiocytoma. However, tumors other than malignant lymphoma and hemangioendothelioma are extremely rare.

Although differentiation between primary splenic tumor and metastatic splenic tumors is difficult based on imaging studies, recent progress in imaging modalities have rendered MRI and PET-CT findings useful. Thymoma with low-grade malignancy shows characteristic uniformity with a low to intermediate signal strength on T1-weighted images and hyperintense signal strength on T2-weighted images. Despite variability, PET-CT scan have shown low accumulation for low-grade thymoma and high accumulation for malignant cases such as thymic cancer, reflecting the level of malignancy. These findings are similarly observed for metastases and are useful for diagnosis. Malignant lymphoma, which requires differentiation, exhibits an intermediate signal intensity on T1-weighted images, intermediate and uniform signal intensity on T2-weighted images, and high accumulation on PET-CT scan. Metastatic malignant tumors often exhibit low signal intensity on T1-weighted images, high signal intensity on T2-weighted images, and high accumulation on PET-CT scan. For benign tumors such as hemangioma and lymphangioma, the tumor appears non-uniform in T1 and T2-weighted images and exhibits low accumulation on PET-CT scan. Images acquired using these modalities are helpful for diagnosis. In the present patient, the findings were not consistent with any of these patterns, making diagnosis difficult to base on imaging data.

In the present patient, the recurrence occurred eight years postoperatively during long-term follow-up. Furthermore, the metastatic tumor showed slow growth. Therefore, we selected surgical resection as the treatment. In the National Comprehensive Cancer Network Guidelines revised in 2014, staging/histological classification and treatment plans for thymoma are summarized in addition to those for thymus cancer [6]. According to these guidelines, complete removal of the tumor is recommended for resectable thymomas, and radiation therapy, chemotherapy, and/or postoperative adjuvant therapy are selected depending on the stage. Although there are reports of splenectomy for isolated splenic metastases from thymic carcinoma, no standardized treatment plans have been proposed [16]. We considered surgical resection to be reasonable because surgical resection is the first-choice treatment for thymoma. Eligibility for and effectiveness of surgical treatment of splenic metastases of malignant tumors vary depending on the primary lesion, and the long-term prognosis is still an issue that requires further investigation. We suggest that long-term follow up for at least 10 years is desirable after thymectomy even if the lesion is a low-grade thymoma [13].

## Conclusions

Thymomas are rare lesions, and extra-thoracic spread of thymomas is extremely uncommon. Isolated splenic metastases from any primary lesion are extremely rare. This is the first report of an isolated splenic metastasis from a thymoma. Further cases are needed to standardize this surgery for such lesions.

## Abbreviations

CT: Computed tomography
MRI: Magnetic resonance imaging
PET-CT: Positron emission tomography-CT
